# Transcriptome analysis of mRNA after different induction time of induced Schwann-like cells from adipose-derived stem cells

**DOI:** 10.1097/JS9.0000000000002716

**Published:** 2025-06-12

**Authors:** Zhongpei Lin, Zeyu Zhang, Qinglin Qiu, Jiamin Lu, Shouwen Su, Chau Wei Wong, Xiaoyue Wen, Bo He

**Affiliations:** a Orthopaedic Trauma and Joint, Department of Orthopedics, The Third Affiliated Hospital of Sun Yat-sen University, Guangzhou, China; b Department of Plastic Surgery, The First Affiliated Hospital of Sun Yat-sen University, Guangzhou, China; c Department of Plastic Surgery, LIHE Hospital, Guangzhou, China

**Keywords:** ADSCs, induction, iSCs, JAK/STAT, transcriptome

## Abstract

**Objectives:**

To compare the differences in protein-coding transcripts before and after different induction times of adipose-derived stem cells (ADSCs)-induced Schwann-like cells (iSCs).

**Methods:**

ADSCs were isolated from healthy adult female rats. In addition, the iSCs of 7 and 19 days after induction were chosen for ribonucleic acid (RNA)-sequencing (RNA-seq). Bioinformatic analysis was applied to determine the differences among ADSCs (group 1, g1), iSCs-7d (group 2, g2), and iSCs-19d (group 3, g3). Eight differentially expressed messenger RNAs (DEmRNAs) were randomly selected for quantitative real-time polymerase chain reaction to verify the accuracy of sequencing data.

**Results:**

Compared with g1, g2, and g3 had 83 and 189 DEmRNAs, respectively. DEmRNAs of g2 were located in the synapse area and enriched in the nucleotide-binding oligomerization domain-like signaling pathway through Kyoto Encyclopedia of Genes and Genomes analysis. Located in the neuromuscular junction and extracellular matrix (ECM), DEmRNAs of g3 were enriched in the cellular response to cyclic adenosine monophosphate and the development of the peripheral nervous system through Gene Ontology analysis. Venn analysis showed that 31 genes were up-regulated after induction, and their protein products were mainly located in the ECM and enriched in the Janus kinase-signal transducer and activator of transcription (JAK/STAT) pathway. Eight genes were found to be down-regulated in these groups. It was discovered that neurofilament high molecular weight (Nefh), neuroregulatory protein 1 (Nrg1), and iodothyronine deiodinase 2 (Dio2) exhibited a persistent increase in quantitative PCR results after induction.

**Conclusion:**

During the induction process of Schwann cells from ADSCs, key regulation factors such as Nefh, Nrg1, and Dio2, as well as the continuous activation of the JAK/STAT pathway may play a role in facilitating cell transdifferentiation.

This study strictly adheres to the TITAN guidelines for experimental design and ethical compliance, with technical frameworks and data validation processes implemented according to the guideline specifications^[1]^.

Peripheral nerve defects present a notable clinical challenge^[2,3]^. Current clinical practice, which relies on the harvest of small cutaneous nerves for the cable-graft reconstruction of peripheral nerve deficits, proves inadequate for addressing large-caliber nerve defects. Donor site morbidity may manifest as surgical scarring, traumatic neuroma, and sensory impairment^[4–6]^. Consequently, ongoing scientific investigation is being conducted into alternative bridging materials for peripheral nerve defects that could potentially supersede autologous nerve grafting, which remains the gold standard at present. Recent advancements, particularly in clinical trials, continue to drive progress in this field^[6–8]^.

Regenerative medicine enables nerve defect repair to get rid of the treatment method of treating trauma with trauma, while acellular tissue matrix (ACTM) has pioneered new pathways for tissue and organ repair and regeneration, and become a focal point in research on biological derivative materials. Currently, allogeneic decellularized nerves are widely used clinically approved natural biological derivative materials like *Shenqiao* in China and *Avance* of AxoGen in the United States^[7]^. However, scaffold materials alone are neither the ideal solution for tissue defect repair nor the ultimate goal of tissue regeneration. To modify ACTM as a primary bridging material, it is necessary to explore novel approaches to enhance its bioactivity and promote nerve regeneration. “Tissue engineering nerves” make it possible, mainly including scaffolds, seed cells, neurotrophic factors, and construction technology^[6,8]^. Closely related to axon regeneration and myelination after peripheral injury, Schwann cells (SCs) are seed cells for constructing tissue engineering nerves^[5,9]^.

In recent years, repairing peripheral nerve injury by planting induced Schwann-like cells (iSCs) directionally differentiated from stem cells has shown wide clinical application. Among them, adipose-derived stem cells (ADSCs) have a number of advantages such as multi-differentiation potential, good proliferation ability, a wide range of sources and low immunogenicity, which has turned into a research hotspot. ADSCs can differentiate into iSCs by inducing differentiation to express SC phenotypes and secrete neurotrophic factors. In this work, ADSCs were successfully induced to differentiate into iSCs in the early stage. It was found that the neurosecretory function of iSCs gradually increased with the extension of induction time, while their proliferation ability decreased. Cell proliferation ability was the best at the early stage of induction, especially within the first 7 days^[10]^. The neurotrophic factor secretion of iSCs was the strongest and tended to be stable at 19 days, but the specific molecular mechanism was unknown^[9,10]^.

Nevertheless, these cells were analyzed for messenger ribonucleic acid (mRNA) sequencing as previous studies did not involve genetic analysis and mechanism investigation^[10]^. Next-generation high-throughput sequencing was performed on iSCs at different induction times, and bioinformatics analysis was conducted to explore the potential mechanism of ADSCs induced into iSCs.

## Statement

The work was reported according to the Animals in Research: Reporting *In Vivo* Experiments guidelines^[11]^.

## Materials and methods

### Materials

**Figure 1.**
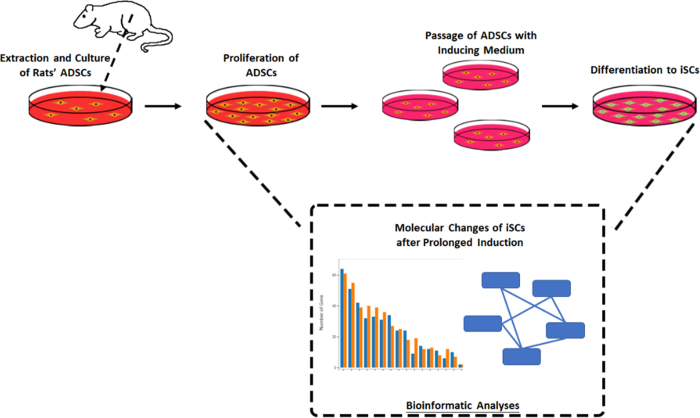
Experimental procedure.

A total of 20 healthy adult female Sprague–Dawley (SD) rats were chosen at random and provided by the Experimental Animal Center, with an average body weight of 200–300 g (Fig. 1).

### Induction and differentiation of iSCs

ADSCs were isolated, purified, and cultured, as mentioned in the previous study^[9]^. After successful isolation, they were kept in a 5% carbon dioxide (CO_2_) and 37°C incubator. The culture medium was changed every 2 days, and cells were passaged when 80% confluency was achieved.

The third generation of ADSCs was gathered and iSCs were induced^[10]^. Cells were subjected to 24-h incubation in a 5% CO_2_ and 37°C incubator by 1 mM 2-mercapto-ethanol (BME; Sigma) in Dulbecco’s Modified Eagle Medium/Nutrient Mixture F-12 (DMEM/F12) (Gibco). After washing with phosphate buffer saline, cells were cultured with 10% fetal bovine serum and 35 ng/ml all-trans-retinoic acid (ATRA; MCE) in DMEM/F12 for another 24 h. Finally, they were kept in 200 ng/ml heregulin (GenScript, China), 5 ng/ml platelet-derived growth factor (PDGF; ProSpec, the United States of America [USA]), 10 ng/ml basic fibroblast growth factor (bFGF; PeproTech, USA), 5 µM forskolin (SIGMA) and 10% FBS in DMEM/F12. The culture medium was changed every other day and cells were passaged when 80% confluency was achieved. A CKX41 optical microscope was used to observe cells 7 and 19 days after induction (Olympus, Japan).

**HIGHLIGHTS**

- Multi-omics reveals dynamic regulatory networks in stem cell differentiation: Through RNA-seq and functional bioinformatics analyses, the transcriptional landscape of adipose-derived stem cells reprogramming into induced Schwann-like cells (iSCs) was uncovered.
- The JAK/STAT pathway is a significant way of controlling the transformation of stem cells into iSCs: The JAK/STAT pathway was demonstrated to not only play a pivotal role in regulating the maintenance of iSCs but also serve as a master coordinator of Schwann cell (SC) differentiation.
- Novel biomarkers for SC function are iodothyronine deiodinase 2 (Dio2), neuroregulatory protein 1 (Nrg1), and molecular weight (Nefh): In this study, Dio2 (thyroid hormone metabolism), Nrg1 (neurotrophic signaling), and Nefh (axon guidance) were identified as critical regulators of SC plasticity. Their coordinated roles in mediating metabolic reprogramming (e.g. mitochondrial oxidative phosphorylation) and axon regeneration were revealed, with Dio2/STAT3 and Nrg1-erythroblastic leukemia viral oncogene homolog (ErbB) axis interactions representing key therapeutic targets for peripheral nerve repair and glioma modeling.

### High-throughput transcriptome sequencing

The transcriptome was compared using next-generation sequencing. All sequencing procedures were conducted by Shanghai Biotechnology Corporation (Shanghai, China). Specific procedures were described in the previous report^[12]^. Briefly, total RNA was extracted with TRIzol, purified and subjected to quality control. In transcriptome sequencing, the number of reads mapped to the gene area showed a positive relationship with the level of gene expression and was also related to the amount of sequencing data and gene length. Therefore, reads were converted into fragments per kilobase of exon per million mapped reads (FPKM) to normalize the level of gene expression^[13]^ and compare the gene expression levels of different genes and samples. The FPKM value of each gene was calculated using Perl scripts, trimmed mean of M values (TMM), and Stringtie (version 1.3.0).

### Bioinformatics and protein interaction network analysis

Differentially expressed (DE) genes were analyzed by edgeR^[14]^. After *P*-values were obtained, multiple hypothesis test correction was performed. The false discovery rate (FDR) was controlled to identify the threshold value for *P*-values^[15,16]^. The *q*-value represented the corrected p-value. In addition, the FPKM value was utilized for calculating the fold differential expression that represented fold change. DE genes were defined to have a *q*-value ≤0.05 and a log_2_FC ≥1. The role of DEmRNAs was determined using Kyoto Encyclopedia of Genes and Genomes (KEGG) pathway and Gene Ontology (GO) analyses. The significance of pathway identifiers and GO terms in the DE gene list was examined by use of Fisher’s exact test. Bioinformatics analysis and RNA sequencing were performed by Shanghai Biotechnology Corporation (Shanghai, China)^[12]^.

The up- and down-regulated DEmRNAs were separately introduced into the online software String (http://string-db.org/) in order to predict the interaction between the translated proteins. The calculated results were introduced into the online analysis platform. In accordance with default settings, the protein-protein interaction network was plotted. Additionally, histograms of up- and down-regulated enriched subgroups and GO and KEGG subgroups were plotted in Excel 2020 (Microsoft, USA) according to the FDR order.

### Quantitative real-time polymerase chain reaction validation of predicted genes

The expression levels of eight DEmRNAs were randomly chosen for verification, including four up-regulated (transforming acidic coiled-coil protein 3 [Tacc3], iodothyronine deiodinase 2 [Dio2], neurofilament high molecular weight [Nefh] and neuroregulatory protein 1 [Nrg1]) and four down-regulated genes (roundabout guidance receptor 2 [Robo2], tissue inhibitor of metalloproteinase 3 [Timp3], periostin [Postn] and atypical chemokine receptor 3 [Ackr3]) at 7 and 19 days after induction using real-time polymerase chain reaction (RT-PCR). The expression of glyceraldehyde-3-phosphate dehydrogenase was chosen as normal control^[12]^. Three replicates were obtained for each specimen. The oligonucleotide sequences of the targeted genes are shown in Table 1. Conventional agarose gel electrophoresis and melting curve analysis were used to confirm the specificity of RT-PCR. The 2− ^ΔΔCt^ method was employed to calculate relative gene expression.

**Table 1:** Primer sequence

Gene name	Forward	Reverse
Tacc3	AAC TGA ACA GGA TCT GCG ATG A	CAG TCA GCT GTG GTC AGA TCT TCT
Timp3	TGT TGA GGT CTT GGC GAG AA	TCT CCA ATG ATG TGA CTT TAC GAA A
Dio2	TGT CCA CCT GTG GTT CTC AGTT	TTC CTG CTG ATA ATC CCC AAA
Ackr3	GCC CAG CAA TGC AGT TTG T	TTG CAG CCC GAA ACG TAA C
Nefh	GCT GGC CGC TGA ACC A	GGA AGC AAT CGA AAG TGG ACT T
Nrg1	TCT GGC TTG CTA TCA GGA TAA ATC T	CCC CCA AGA CAG AAA GTG AAA C
Postn	GCC AAG CCA CCA AAT TAC AAA	GCA GGA AAC CCA CAT TGC A
Robo2	AAT AGA TCC TGT GTC ACT TTC AGA ATT C	TCC TAC TTA GCA CTT CTG ATC CAT ATT T
Gapdh	TGA CTT CAA CAG CGA CAC CCA	CAC CCT GTT GCT GTA GCC AAA

### Data collection and statistical analyses

Collected experimental data were expressed as mean ± standard deviation (SD). All data were introduced into SPSS 23.0 (Statistical Package for the Social Sciences Inc., USA). The differences among groups were analyzed by analysis of variance. The least significant difference method was adopted to validate pair-wise differences between the two groups. The significance level was set to *α* = 0.05.

## Results

### Preprocessing results and transcriptome sequencing

After the complementary deoxyribonucleic acid library of sequencing samples was constructed, RNA sequencing was conducted via the Illumina HiSeq 2500 platform. The preprocessing results of the raw reads of each sample are presented in Table 2. The results indicated that the clean read ratio of all samples ranged from 91.94% to 96.42%, which suggested that all the data were reliable and high-quality. After the removal of reads with only single-end sequencing and ribosomal RNA reads, pre-mapped reads were matched to find the mapped reads of rat genome sequencing. Most samples had a mapping ratio of 90% except for ADSCs 3 (75.28%), ADSCs 4 (63.28%), and iSCs 7d3 (79.53%). The ratio of the unique mapped reads of all samples reached 99%.

**Table 2:** Top 10 up-/down-regulated DEmRNAs in different groups (iSC 7d vs. ADSC)

Gene ID	Gene name	ADSC-FPKM	iSC7d-FPKM	log2FC	Q_value
Up-regulated genes
ENSRNOG00000002578	Serpinb3a	0	9.1372	Inf	0.0103
ENSRNOG00000029760	AABR07012291.1	0	26.1504	Inf	0.0288
ENSRNOG00000017609	Cnga4	0	0.3744	Inf	0.0372
ENSRNOG00000029508	Slpil2	0	8.7019	Inf	0.0476
ENSRNOG00000055916	AABR07012301.1	0.0782	72.8975	9.8639	0.0136
ENSRNOG00000009054	Elmod1	0.0061	1.2916	7.7283	0.0308
ENSRNOG00000015957	F13a1	0.0307	4.5060	7.1973	0.0043
ENSRNOG00000002460	Serpinb2	0.2247	22.8955	6.6707	0.0193
ENSRNOG00000055103	Cftr	0.0116	1.1432	6.6277	0.0136
ENSRNOG00000003189	Cited1	0.2481	24.2876	6.6133	0.0078
Down-regulated genes
ENSRNOG00000005800	Il1f10	2.862285	0	N/A	0.004287
ENSRNOG00000051257	Rn60_X_0752.3	58.66133	0.049927	−10.1984	0.013554
ENSRNOG00000049880	Tmem200c	0.668077	0.007001	−6.57623	0.004083
ENSRNOG00000056314	AABR07056330.1	1.281237	0.02033	−5.97782	0.043718
ENSRNOG00000043465	Arc	0.821539	0.015326	−5.74426	0.025529
ENSRNOG00000004540	LOC100912012	9.401489	0.26611	−5.14279	0.025529
ENSRNOG00000048924	Islr	6.45329	0.346231	−4.22023	0.000317
ENSRNOG00000004275	Egfl6	70.62361	4.858477	−3.86157	0.035368
ENSRNOG00000015055	Scg2	2.990841	0.210758	−3.82689	0.047595
ENSRNOG00000004476	Wif1	4.892156	0.370043	−3.72471	0.008795

Inf, infinity; N/A, not applicable.

### GO/KEGG analysis of the effect of early induction (7 days) on iSCs

**Figure 2.**
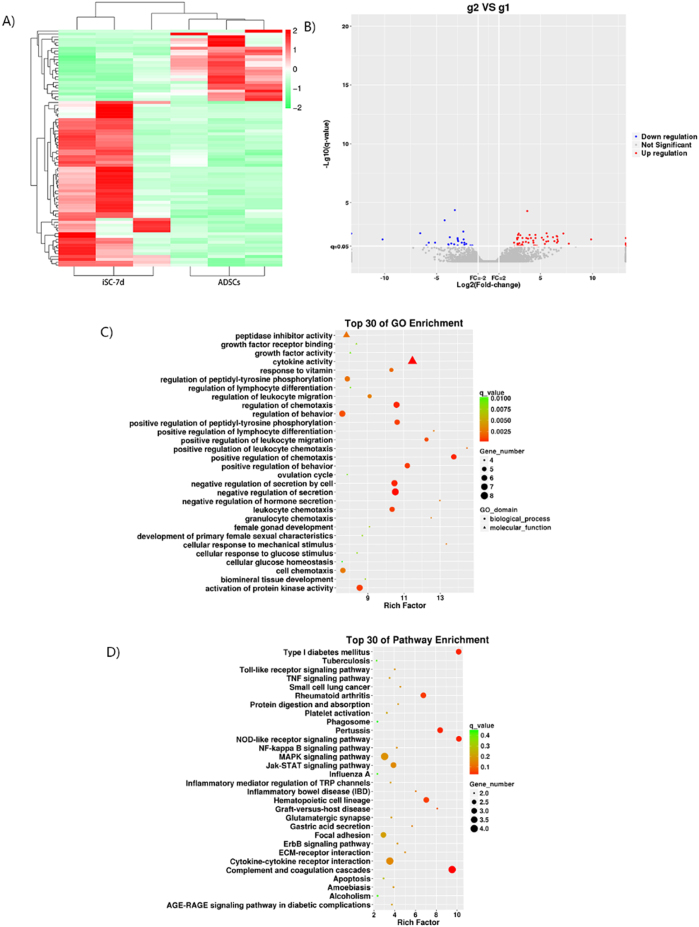
Differential mRNA profile between cells (iSCs-7d, g2) at the stage of early induction and ADSCs (g1). (A) Cluster analysis thermogram of DEmRNAs between iSCs-7d and ADSCs, in which the red gene indicates up-regulation in iSCs-7d and the blue gene indicates down-regulation in iSCs-7d. (B) Volcanic plot of DEmRNAs between iSCs-7d and ADSCs, in which red denotes the up-regulation of gene expression in iSCs-7d, blue denotes the down-regulation of gene expression in iSCs-7d, and gray denotes no difference in expression between the two groups. (C) GO enrichment bubble chart of DEmRNAs between iSCs-7d and ADSCs. Different shapes represent different categories. For example, triangles represent molecular functions and circles stand for BPs. A greater number of DEmRNAs is indicated by a larger shape area. Different colors indicate significant differences in the distribution of DEmRNAs in different subcategories between the two groups, where red, orange, yellow, and green indicate *q* < 0.0025, *q* = 0.0025–0.005, *q* = 0.005–0.0075 and *q* > 0.0075, respectively. The test criterion was 0.05. (D) KEGG pathway enrichment bubble chart of DEmRNAs between iSCs-7d and ADSCs. A higher number of DEmRNAs is indicated by a larger graphical area. Different colors indicate significant differences in the distribution of DEmRNAs in different channels between the two groups, where red, orange, yellow, and green indicate *q* < 0.1, *q* = 0.1–0.2, *q* = 0.2–0.3, and *q* > 0.3, respectively. The test criterion was 0.05.

The transcriptome analysis of iSCs at 7 days after induction showed that compared with those before induction, 83 groups of DEmRNAs appeared on iSCs-7d. Among them, 58 groups were up-regulated, such as serpin peptidase inhibitor, clade B (ovalbumin), member 3 (Serpinb3a), cyclic nucleotide-gated channel alpha-4 (Cnga4), Slpil2, cystic fibrosis transmembrane conductance regulator (Cftr) and carboxy-terminal domain, 1 (Cited1), and 25 groups were down-regulated, such as interleukin-1 family member 10 (Il1f10), transmembrane protein 200C (Tmem200c) and activity-regulated cytoskeleton-associated gene (Arc) (Tables 3 and 4. Fig. 2A and B).

**Table 3:** GO functional enrichment of DEmRNAs to the ADSC vs. iSC-7d

GO_ID	GO_term	RF	Q_value	UP_list	DOWN_list
Cellular Component
GO:0014069	Postsynaptic density	5.6540	0.0248	Nefh, Bsn	Ntrk2, Arc
GO:0060076	Excitatory synapse	5.2950	0.0300	Nefh, Bsn	Ntrk2, Arc
GO:0005615	Extracellular space	5.2696	0.0000	Hpx, Fam132b, Nrg1, Ucn2, F13a1, Thbd, Ereg, Il23a, Il1b, Serpinb2, Serpinb3a, Col7a1, Spp1, Itga2	Nucb2, Bmp3, Scg2, Il1f10, Cpxm1, Chrd, LOC100912012, Gas6, Sepp1
GO:0030424	Axon	4.5608	0.0093	Tpx2, Nefh, Bsn, Itga2, Nrg1	Robo2, Ntrk2
GO:0045177	Apical part of cell	3.8970	0.0470	Spp1, Nrg1, Cftr, Slc1a1	Slc29a1
Biological Process
GO:0002690	Positive regulation of leukocyte chemotaxis	14.5038	0.0021	Cxcl14, Il1b, Il23a	Gas6
GO:0050921	Positive regulation of chemotaxis	13.7719	0.0003	Il1b, Cxcl14, Itga2, Il23a	Gas6, Scg2
GO:0071260	Cellular response to mechanical stimulus	13.3435	0.0025	Il1b, Il13ra2, Itga2	Gadd45a
GO:0046888	Negative regulation of hormone secretion	12.9969	0.0026	Il11, Ucn2, Il1b	Nucb2
GO:0045621	Positive regulation of lymphocyte differentiation	12.6678	0.0029	Il23a, Nlrp3, Il1b	Gas6
Molecular Function
GO:0005125	Cytokine activity	11.5030	0.0001	Il11, Il23a, Cxcl14, Il1b, Spp1	Il1f10, Bmp3, Scg2
GO:0070851	Growth factor receptor binding	8.4097	0.0083	Ereg, Il1b, Il11	Il1f10
GO:0008083	Growth factor activity	8.0706	0.0091	Ereg, Il11	Gas6, Bmp3
GO:0030414	Peptidase inhibitor activity	7.8594	0.0025	Serpinb2, Serpinb3a, Birc3, Slpil2, Slpi	Gas6
GO:0005126	Cytokine receptor binding	6.3878	0.0050	Il11, Il23a, Cxcl14, Il1b	Il1f10, Nucb2
GO:0004866	Endopeptidase inhibitor activity	5.8524	0.0221	Birc3, Serpinb3a, Serpinb2	Gas6
GO:0004857	Enzyme inhibitor activity	4.6331	0.0148	Serpinb3a, Slpil2, Serpinb2, Birc3, Slpi	Gas6
GO:0005102	Receptor binding	4.0137	0.0001	Il11, Cxcl14, Socs2, Itga2, Hmga1, Spp1, RT1-CE4, Ucn2, Nrg1, Fam132b, Il1b, Il23a, Ereg	Gas6, Egfl6, Ntrk2, Nucb2, Il1f10, Bmp3, Cpe, Scg2

RF, richment factor.

**Table 4:** KEGG enrichment of DEmRNAs to the ADSC vs. iSC-7d

ID	Pathway description	RF	*Q*_value	UP_list	DOWN_list
rno04621	NOD-like receptor signaling pathway	10.2148	0.0315	Il1b,Birc3, Nlrp3	–
rno04940	Type I diabetes mellitus	10.2148	0.0315	Il1b, RT1-CE4	Cpe
rno04610	Complement and coagulation cascades	9.5338	0.0218	Serpinb2, Thbd, Procr, F13a1	–
rno05133	Pertussis	8.4122	0.0324	Il23a, Il1b, Nlrp3	–
rno04640	Hematopoietic cell lineage	7.0621	0.0491	Il11, Itga2, Il1b	–
rno05323	Rheumatoid arthritis	6.8098	0.0466	Il23a, Il11, Il1b,	–

RF, richment factor.

The GO analysis of all DEmRNAs between iSCs-7d and ADSCs showed that DEmRNAs at 7 days after induction were mainly located at the postsynaptic density (ratio frequency [RF] = 5.654, *q* < 0.01), excitatory synapse (RF = 5.295, *q* < 0.01), extracellular matrix (ECM) (RF = 5.270, *q* < 0.01) and axon (RF = 4.561, *q* < 0.01). In biological processes (BPs), they were mainly enriched in the positive regulation of leukocyte chemotaxis (RF = 14.504, *q* < 0.01) and chemotaxis (RF = 13.772, *q* < 0.01), the cellular response to mechanical stimuli (RF = 13.343, *q* < 0.01), the negative regulation of hormone secretion (RF = 12.997, *q* < 0.01) and the positive regulation of lymphocyte differentiation (RF = 12.668, *q* < 0.01). These DEmRNAs mainly performed cytokine activity (RF = 11.503, *q* < 0.01), growth factor receptor binding (RF = 8.410, *q* < 0.01), growth factor activity (RF = 8.071, *q* < 0.01), peptidase inhibitor activity (RF = 7.859, *q* < 0.01) and cytokine receptor binding (RF = 6.388, *q* < 0.01) (Fig. 2C).

KEGG analysis found that the involved pathways included the nucleotide-binding oligomerization domain-like receptor signaling pathway (RF = 10.215, *q* < 0.05), type I diabetes mellitus (RF = 10.215, *q* < 0.05), complement and coagulation cascades (RF = 9.534, *q* < 0.05), pertussis (RF = 8.412, *q* < 0.05), hematopoietic cell lineage (RF = 7.062, *q* < 0.05) and rheumatoid arthritis (RF = 6.810, *q* < 0.05) (Fig. 2D).

### GO/KEGG analysis of the effect of late induction (19 days) on iSCs

**Figure 3.**
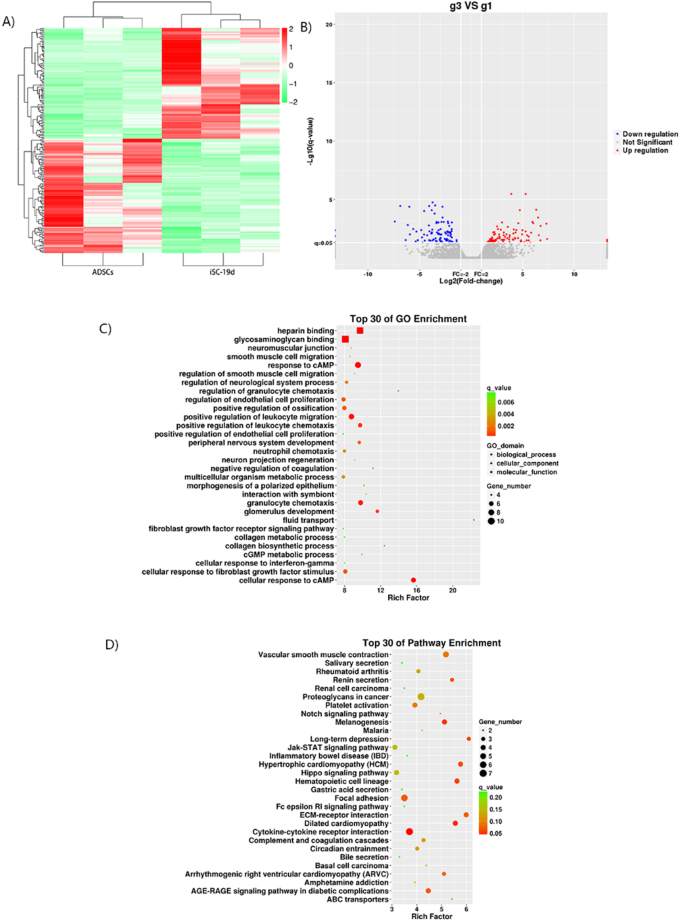
Differential mRNA profile between cells (iSCs-19 d, g3) at the stage of late induction and ADSCs (g3). (A) Cluster analysis thermogram of DEmRNAs between iSCs-19d and ADSCs, in which red indicates up-regulation in iSCs-19d and blue indicates down-regulation in iSCs-19d. (B) Volcanic plot of DEmRNAs between iSCs-19d and ADSCs, in which red denotes the up-regulation of gene expression in iSCs-19d, blue denotes the down-regulation of gene expression in iSCs-19d, and gray denotes no difference in expression between the two groups. (C) GO enrichment bubble chart of DEmRNAs between iSCs-19d and ADSCs. Different shapes represent different categories. For example, triangles represent molecular functions and circles stand for BPs. A greater number of DEmRNAs are indicated by a larger shape area. Different colors indicate significant differences in the distribution of DEmRNAs in different subcategories between the two groups, where red, orange, yellow, and green indicate *q* < 0.002, *q* = 0.002–0.004, *q* = 0.004–0.006 and *q* > 0.006, respectively. The test criterion was 0.05. (D) KEGG pathway enrichment bubble chart of DEmRNAs between iSCs-19d and ADSCs. A higher number of DEmRNAs is indicated by a larger graphical area. Different colors indicate significant differences in the distribution of DEmRNAs in different channels between the two groups, where red, orange, yellow, and green indicate *q* < 0.1, *q* = 0.1–0.15, *q* = 0.15–0.2 and *q* > 0.2, respectively. The test criterion was 0.05.

The transcriptome analysis of iSCs at 19 days after induction showed that compared with those before induction, 189 groups of DEmRNAs appeared on iSCs-19d. Among them, 93 groups were up-regulated, such as ethanolamine-phosphate phospho-lyase (Etnppl), Slpil2, adhesion G protein-coupled receptor E1 (Adgre1), Cftr and Cited1, and 96 groups were down-regulated, such as calsequestrin 2 (Casq2), membrane frizzled-related protein (Mfrp), Nel-related protein 2 (Nell2) and Arc (Table 5; Fig. 3A and B).

**Table 5:** Top 10 up-/down-regulated DEmRNAs in different groups (iSC-19d vs. ADSC)

Gene ID	Gene name	ADSC-FPKM	iSC19d-FPKM	log2FC	Q_value
Up-regulated genes
ENSRNOG00000045743	Etnppl	0	0.1513	Inf	0.0277
ENSRNOG00000029508	Slpil2	0	5.3317	Inf	0.0344
ENSRNOG00000046254	Adgre1	0	0.3609	Inf	0.0420
ENSRNOG00000009054	Elmod1	0.0061	1.0086	7.3715	0.0242
ENSRNOG00000055103	Cftr	0.0116	1.8235	7.3013	0.0012
ENSRNOG00000013496	Crisp3	0.1499	16.0650	6.7433	0.0019
ENSRNOG00000023126	Rxfp3	0.0210	2.2205	6.7219	0.0323
ENSRNOG00000013981	Ptpn5	0.0238	2.2546	6.5677	0.0117
ENSRNOG00000032973	Il13ra2	0.0919	8.3070	6.4984	0.0004
ENSRNOG00000003189	Cited1	0.2481	19.7297	6.3134	<0.001
Down-regulated genes
ENSRNOG00000060440	AABR07055333.1	1.3543	0	N/A	0.0044
ENSRNOG00000016243	Casq2	0.4848	0	N/A	0.0140
ENSRNOG00000039107	Mfrp	0.1506	0	N/A	0.0474
ENSRNOG00000006235	Nell2	0.9046	0.0054	−7.3934	0.0008
ENSRNOG00000029911	Cilp	12.9431	0.1112	−6.8625	<0.001
ENSRNOG00000060970	Tnmd	1.1183	0.0138	−6.3449	0.0306
ENSRNOG00000029401	Actg2	5.9757	0.0848	−6.1381	0.0016
ENSRNOG00000010666	Wisp2	169.5984	2.6660	−5.9913	<0.001
ENSRNOG00000043465	Arc	0.8215	0.0135	−5.9221	0.0099
ENSRNOG00000021735	Akr1cl	3.1863	0.0654	−5.6072	0.0034

Inf, infinity; N/A, not applicable.

The GO analysis of all DEmRNAs between iSCs-19d and ADSCs showed that DEmRNAs at 19 days after induction were mainly located at the neuromuscular junction (RF = 8.758, *q* < 0.01), blood microparticle (RF = 7.614, *q* < 0.01), ECM (RF = 6.240, *q* < 0.01), sarcolemma (RF = 5.982, *q* < 0.01) and spindle pole (RF = 5.528, *q* < 0.05). In BPs, they were enriched in the cellular response to cyclic adenosine monophosphate (cAMP) (RF = 15.633, *q* < 0.01), collagen biosynthetic process (RF = 12.407, *q* < 0.05), cGMP metabolic process (RF = 9.926, *q* < 0.01), peripheral nervous system development (RF = 9.626, *q* < 0.01), response to cAMP (RF = 9.481, *q* < 0.01), neuron projection regeneration (RF = 9.116, *q* < 0.01), etc. These DEmRNAs mainly performed heparin-binding (RF = 9.710, *q* < 0.01), glycosaminoglycan binding (RF = 8.081, *q* < 0.01), growth factor activity (RF = 7.204, *q* < 0.01), integrin binding (RF = 6.727, *q* < 0.01) and cytokine activity (RF = 5.134, *q* < 0.01) (Tables 6 and 7. Fig. 3C and D).

**Table 6:** GO functional enrichment of DEmRNAs to the ADSC vs. iSC-19d

	GO_ID	GO_term	RF	*Q*value	UP_list	DOWN_list
Cellular Component	GO:0031594	Neuromuscular junction	8.7582	0.0055	Serpine2, Itga7, Nrg1	Postn
	GO:0072562	Blood microparticle	7.6136	0.0013	Hpx, F13a1	Apoe, Actg2, Stom, Acta1
	GO:0031012	Extracellular matrix	6.2402	0.0000	Col7a1, Wnt5a, Mmp12, Serpine2, Mmp10, Lama5	Apoe, Ctgf, Postn, Egflam, Mfap5, Tgfb3, Cilp, Rarres2, Cthrc1, Pcsk6, Cpxm2, Timp3, Adamts5
	GO:0042383	Sarcolemma	5.9821	0.0041	Itga7	Pgm5, Kcnd3, Aqp1, Sgcg, Tgfb3
	GO:0000922	Spindle pole	5.5281	0.0117	Tpx2, Cenpf, Aspm, Kif11, Kif20b	–
Molecular Function	GO:0008201	Heparin-binding	9.7101	0.0000	Serpine2	Pcsk6, Gpnmb, Chrd, Mdk, Nell2, Postn, Ctgf, LOC100912012, Apoe
	GO:0005539	Glycosaminoglycan binding	8.0811	0.0000	Serpine2	Apoe, Postn, Ctgf, Pcsk6, LOC100912012, Mdk, Nell2, Egflam, Gpnmb, Chrd
	GO:0008083	Growth factor activity	7.2043	0.0003	Hgf, Kitlg, Il11	Mdk, Ctgf, Gas6, Clec11a, Tgfb3
	GO:0005178	Integrin binding	6.7269	0.0057	Itga7, Lama5, Itga2	Ctgf, Gpnmb
	GO:0005125	Cytokine activity	5.1341	0.0021	Il11, Il23a, Kitlg, Wnt5a, Spp1	Edn1, Tgfb3, Ccl11
Biological Process	GO:0042044	Fluid transport	22.3333	0.0002	Slc14a1	Itpr1, Aqp1, Edn1
	GO:0071320	Cellular response to cAMP	15.6333	0.0000	Cftr, Penk	Nox4, Egr3, Itpr1, Aqp1, Egr2
	GO:0071622	Regulation of granulocyte chemotaxis	13.9583	0.0011	Il23a	Edn1, Rarres2, Tnfsf18
	GO:0032964	Collagen biosynthetic process	12.4074	0.0016	Itga2	Tgfb3, Ctgf, Cygb
	GO:0032835	Glomerulus development	11.6319	0.0006	–	Sulf2, Jag1, Aqp1, Plce1, Notch3
Key biological items	GO:0051702	Interaction with symbiont	10.3876	0.0029	–	Apoe, Ptx3, Aqp1, Stom
	GO:0001738	Morphogenesis of a polarized epithelium	10.1515	0.0032	Wnt5a, Lama5	Fzd6, Cthrc1
	GO:0046068	cGMP metabolic process	9.9259	0.0035	Wnt5a	Aqp1, Apoe, Gucy1b3
	GO:0007422	Peripheral nervous system development	9.6264	0.0013	Fa2h, Nrg1, Nefh	Egr2, Egr3
	GO:0051591	Response to cAMP	9.4811	0.0000	Thbd, Cited1, Penk, Cftr	Itpr1, Egr2, Aqp1, Nox4, Egr3
	GO:0031102	Neuron projection regeneration	9.1156	0.0047	Spp1, Nefh, Hgf	Apoe

RF, richment factor.

**Table 7:** KEGG enrichment of DEmRNAs to the ADSC vs. iSC-19d.

ID	Pathway description	RF	*Q*_value	UP_list	DOWN_list
rno05414	Dilated cardiomyopathy	5.5507	0.0492	Itga2, Itga7	Sgcg, Tgfb3
rno04060	Cytokine-cytokine receptor interaction	3.7048	0.0426	Kitlg, Il11, Il23a, Hgf	Tgfb3, Ackr3, Ccl11

RF, richment factor.

### Intersection analysis of DEmRNAs at different induction times

**Figure 4.**
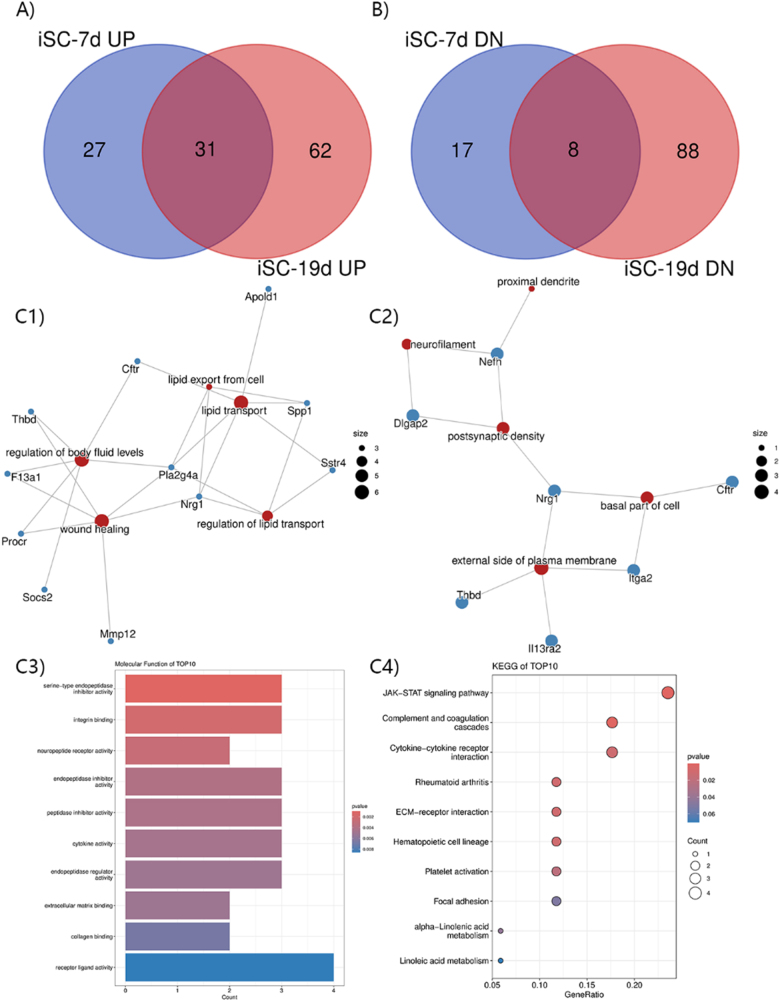
Venn diagram of DEmRNAs with increased or decreased expression at the intersection of iSCs-7d or iSCs-19d compared with ADSCs. (A) Up-regulated DEmRNAs after induction. (B) Down-regulated DEmRNAs after induction. (C1–C4) Functional enrichment of shared genes up-regulated in both iSCs-7d and iSCs-19d, including (C1) BP, (C2) cellular component, (C3) molecular function and (C4) KEGG enrichment analysis. The test criterion was 0.05.

DEmRNAs at different induction times were analyzed by intersection analysis to find the potential mechanism of inducing iSCs. The results showed that 31 groups of up-regulated genes of iSCs-7d intersected with the up-regulated genes of iSCs-19d (Fig. 4A). The analysis also found the main enrichment of these genes in the neurofilament, proximal dendrite, and postsynaptic density. They executed functions regarding lipid transport and export, the regulation of lipid transport and wound healing, and neuropeptide receptor activity (Fig. 4C1–C3). They affected the activation of the Janus kinase-signal transducer and activator of the transcription (JAK/STAT) signaling pathway (Fig. 4C4). Among the down-regulated genes of iSCs-7d and iSCs-19d, eight groups of genes intersected with others, mainly including Arc, Robo2, Selenoprotein P (Selenop), C-type lectin domain family 3 member B (Clec3b), etc (Fig. 4B).

### Expression of DEmRNAs at different induction times by qRT-PCR

**Figure 5.**
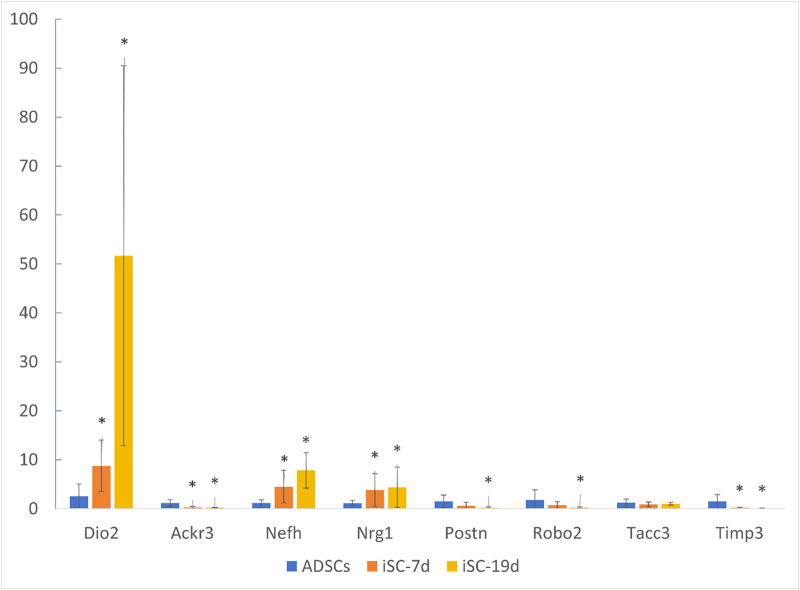
qRT-PCR validation of DEmRNAs, including four up-regulated genes (Tacc3, Dio2, Nefh and Nrg1) and four down-regulated genes (Robo2, Timp3, Postn and Ackr3). Different colors indicate different groups, where blue, red, and green denote ADSCs (g1), iSCs-7d (g2), and iSCs-19 (g3), respectively. Compared to the control group, * indicates *P* < 0.05 and ** indicates *P* < 0.01.

Eight groups of DEmRNA were randomly selected from the sequencing results for quantitative PCR (qPCR) verification. The results showed that the expression trends of Nefh, Nrg1, and Postn were basically consistent with the sequencing results. Nefh and Nrg1 both demonstrated a significant increase at iSCs-7d and iSCs-19d, while Postn decreased with the extension of induction time. However, the difference was significant only at iSCs-19d. The qPCR expression of some genes was not completely in line with the trend of the sequencing results. For example, TIMP3, Ackr3, and Robo2 were down-regulated after induction. The former two genes showed significant statistical differences on the 7th and 19th days of induction, whereas the latter one only showed significant differences on the 19th day of induction. Dio2 was up-regulated continuously after induction, especially at 19 days after induction (Fig. 5).

## Discussion

SC transplantation is an important strategy for treating peripheral nerve injury diseases. SC transplantation capable of promoting axon regeneration and myelination holds significant promise for repairing peripheral nerve injury using biological tissue engineering nerve grafts^[17]^. However, autologous SC extraction faces challenges analogous to autologous nerve transplantation^[18]^. Furthermore, the isolation and purification of autologous SCs are substantially constrained by their limited proliferative capacity and contamination with fibroblasts in autologous nerve extracts^[18]^.

Recent studies have demonstrated that autologous cells from diverse sources can differentiate into SCs. These cells not only exhibit SC phenotypes and secrete neurotrophic factors but also circumvent the technical limitations associated with direct SC extraction. Such SC transplantation offers a novel strategy for developing biological tissue engineering nerve grafts and treating peripheral nerve injury^[19]^. Current evidence confirms that various stem cell types^[20,21]^ can be differentiated into SCs, and provide a scalable and clinically relevant cell source for regenerative therapies^[22]^. These stem cell types encompass bone marrow mesenchymal stem cells (BMSCs), adipose mesenchymal stem cells (ADSCs), umbilical cord mesenchymal stem cells, hair follicle/skin stem cells, and pluripotent stem cells

With many advantages such as a wide range of sources, good proliferation ability, low immunogenicity, and multidirectional differentiation potential, adipose-derived mesenchymal stem cells are a good choice for seed cells^[23]^. It is reported that different induction methods promote the differentiation of adipose-derived mesenchymal stem cells into SCs, and some shortcomings of iSCs are also detected. The differentiation of iSCs requires continuous induction. After the withdrawal of induction factors, SCs cannot maintain their phenotypes or secrete neurotrophic factors, which indicates the reversibility of the differentiation process^[24]^.

Previous studies revealed significant functional dynamics during the differentiation of ADSCs into iSCs: the proliferation capacity of iSCs progressively declined with the extension of induction time (≥7 days) and reached less than 50% of pre-induction levels by day 19. Concurrently, the expression of myelin basic protein (MBP), a marker associated with the differentiation of iSCs, exhibited a >2.5-fold increase on day 19, which indicated the functional maturation of iSCs^[9]^. Notably, this functional transition exhibited stage-specific characteristics: iSCs maintained optimal proliferation and viability during early induction (≤7 days), but demonstrated enhanced neurotrophic factor secretion capacity (e.g. a 3.8-fold increase in brain-derived neurotrophic factor [BDNF] secretion) during late-stage induction (≥14 days).

Nonetheless, the molecular regulatory network that governs the differentiation of ADSCs into iSCs remains poorly understood. Therefore, it has become a pressing scientific challenge in regenerative medicine to elucidate the molecular determinants of the functional plasticity of iSCs and develop engineered iSC systems with controlled temporal differentiation kinetics.

JAK/STAT pathway can regulate the differentiation of iSCs

Combined with previous research^[9,10]^, it can be seen that ADSCs can be transformed into iSCs with the morphological and biological functions of SCs after pre-induction with BME and ATRA combined with induction with forskolin, bFGF, PDGF, and heregulin. The functional enrichment analysis of DEmRNAs and iSCs induced in different stages showed that the proteins encoded by induced differential genes mainly gathered in synapses, axons, and the ECM, and performed peripheral nerve development and axon regeneration through binding with integrins and growth factors and cAMP-related reactions. Further cross-analysis of DEmRNAs demonstrated that the proteins corresponding to the up-regulated genes mainly gathered in the ECM and performed the regulation of lipid transport, stress response, and cell secretory function during the whole induction process. KEGG enrichment analysis suggested that the activation of the JAK/STAT signaling pathway was significantly associated with the transformation of iSCs.

The JAK/STAT signaling pathway is a major transcription factor and mitochondrial activator necessary for nerve development and regeneration^[25]^. Increasingly considered a very important growth factor and cytokine signaling mechanism in mammals, it plays a vital role in maintaining the normal function of nerve tissue, facilitating the regeneration of damaged nerves and promoting the proliferation, migration, apoptosis, and autophagy of glial cells^[26]^. Moreover, the JAK/STAT pathway also gets involved in the differentiation of oligodendrocytes (OLs), which is closely linked to the formation of myelin sheath. Steelman *et al*^[27]^ found that the activation of STAT3 in OLs can promote cell regeneration, myelination, and remyelination. Dey *et al*^[28]^ noticed that the JAK/Stat cell pathway can affect the differentiation and maturation of OLs by inducing the differentiation of neural stem cells from human embryos, and blocking the phosphorylation of STAT3 will significantly reduce the number of MBP-positive cells. More than that, JAK/STAT is also of importance to maintain the phenotype of astrocytes and microglia in the central nervous system. Magistri *et al*^[29]^ noted that the combination of ciliary neurotrophic factor and bone morphogenetic protein 4 can activate both JAK/STAT and SMAD signaling pathways, inhibit the transformation of OL cell lines and promote the differentiation of astrocytes.

In the peripheral nervous system, the JAK/STAT pathway has the capacity to affect the proliferation, migration, and myelination of SCs^[30]^. Duan *et al*^[25]^ discovered that the mutation of STAT3 or the inhibition of the JAK/STAT pathway can delay axon regeneration in zebrafish, and the migration of SCs in mutant animals is also obviously inhibited. Wu *et al*^[26]^ found that the overexpression of runt-related transcription factor 3 (Runx3) in SCs can activate the JAK/STAT signaling pathway, and then regulate SC proliferation and myelination. Lin *et al*^[31]^ observed that BDNF can play a role by activating the JAK/STAT pathway of SCs. Thus, cells can continuously secrete cytokines and finally promote the regeneration of peripheral nerves. Bressy *et al*^[32]^ revealed that activating the JAK/STAT3/AKT signaling pathway in pancreatic ductal adenocarcinoma models promotes the differentiation of neural glial SCs. The JAK/Stat pathway also plays an essential role in the transdifferentiation of ADSCs. Through comparing the osteogenic effects of cells from different sources, Gou *et al*^[33]^ detected that mouse ADSCs showed the highest transformation efficiency, which was primarily achieved through the activation of the JAK/STAT pathway. It was postulated that the JAK/STAT pathway modulates the phenotypic transitions and biological behaviors of ADSCs during induction, and drives iSCs to adopt a more mature SC-like state with prolonged induction. Mechanistically, this pathway orchestrates the adaptive reprogramming of SCs by regulating mitochondrial oxidative phosphorylation levels^[34]^. Moreover, JAK/STAT signaling drives cell cycle progression via the STAT3 phosphorylation-mediated transcription of genes (e.g. cyclin D1 and myelocytomatosis oncogene), while synergizing with the PI3K/AKT pathway to regulate cell survival and functional differentiation^[35]^. Recent advances highlight the crosstalk between JAK/STAT and other pathways (e.g. transforming growth factor-beta and mitogen-activated protein kinase) in fine-tuning cellular responses, which further underscores the therapeutic potential of JAK/STAT in nerve regeneration.

## Potential key genes regulating the transformation of iSCs

In addition to the JAK/STAT pathway, several key genes, including Nefh, Nrg1, and Dio2, were identified to play a crucial role in maintaining the traits of iSCs. These genes might further enhance the understanding of the molecular mechanisms driving the differentiation of iSCs. It was found that Nefh, Nrg1, and Dio2 genes were continuously up-regulated with the extension of time, which signified that these genes may play a very significant role in maintaining the traits of iSCs.

Nefh, an important cytoskeleton protein in neuron axons, can maintain the morphological stability of axons and ensure the realization of neuron signal transduction function. The protein encoded by Nefh can be used as a marker for nerve injury and regeneration^[36]^. Proteins encoded by Nrg1 belong to one of the four proteins of the neuregulin family, which mainly acts on the receptors of the epidermal growth factor receptor family. Nrg1 has the ability to regulate the proliferation, migration, and myelination of SCs by binding with the ErbB2/3 receptor. At the early development stage of SCs, Nrg1/ErbB can promote the proliferation of SC precursors and stimulate their migration along axons. Then, Nrg1/ErbB can promote the formation of myelin sheath around axons at the mature stage of SCs^[37,38]^. After acute nerve injury, Nrg1 can participate in the process of nerve repair and myelin regeneration^[39]^, but its continuous high expression will influence the function of SCs and induce the occurrence of peripheral neuropathy. Yang *et al*^[40]^ found that the Nrg1/ErbB pathway can promote the differentiation of BMSCs into SCs and play a critical role in SC proliferation and migration. Dio2 is an endoplasmic-reticulum retention protein, which is a marker of astrocyte maturation in the central nervous system^[41]^. Distributed in cortical radial glial cells^[42]^, OLs^[43]^, microglia^[44]^, neural stem cells^[45]^, and retinal Muller glial cells^[46]^, Dio2 is involved in the development, maturation, and photosensitivity of the nervous system and other physiological processes. It activates thyroid hormones^[46]^ by transforming protothyroxine (T4) into bioactive 3,3ʹ,5-triiodothyronine (T3) via outer ring deiodination. T3 usually exerts its function through the thyroid nuclear receptor (TR). The TR can promote the development of neuron cytoskeleton, the differentiation and maturation of neural stem cells, and the formation of synapses. It also promotes the proliferation of glial cells and the formation of myelin sheath^[44,47]^, induces the synthesis of nerve growth factors and enzymes, and propels the development of neuron skeleton^[42]^. From the interaction network constructed in this study, it was speculated that during the induction of iSCs, the elevation of Nefh, Nrg1, and Dio2 can promote the formation and maintenance of cytoskeleton proteins of iSCs, make iSCs secrete numerous myelin-related proteins and neurotrophic factors, and prompt iSCs to differentiate into more mature cell phenotypes through JAK/STAT, Nrg1/ErbB and thyroid hormone-related pathways.

While the differentiation of ADSCs into iSCs has been extensively reported, the present study constitutes neither a reiteration of this cellular reprogramming process nor a mere replication of established protocols. This investigation specifically interrogates the dynamic expression profiles of pivotal mRNAs governing the transdifferentiation of ADSCs into iSCs and thereby provides substantive advancement in understanding the molecular mechanism underlying this phenotypic conversion. This study preliminarily reveals the JAK/STAT pathway and key gene regulatory networks. Subsequent research needs to further investigate the role of sustained JAK/STAT pathway activation in facilitating cellular transdifferentiation, which provides novel molecular targets for the repair of peripheral nerve injury through stem cell therapy^[48]^.

## Conclusion

During the induction process of SCs from ADSCs, key regulation factors such as Nefh, Nrg1, and Dio2, as well as the continuous activation of the JAK/STAT pathway may play a part in helping cell transdifferentiation.

## Data Availability

Not applicable.
